# Quantitative trait loci for plant height in Maresi × CamB barley population and their associations with yield-related traits under different water regimes

**DOI:** 10.1007/s13353-016-0358-1

**Published:** 2016-07-22

**Authors:** Krzysztof Mikołajczak, Anetta Kuczyńska, Paweł Krajewski, Aneta Sawikowska, Maria Surma, Piotr Ogrodowicz, Tadeusz Adamski, Karolina Krystkowiak, Andrzej G. Górny, Michał Kempa, Iwona Szarejko, Justyna Guzy-Wróbelska, Kornelia Gudyś

**Affiliations:** 1 Institute of Plant Genetics, Polish Academy of Sciences, Strzeszyńska 34, 60-479 Poznań, Poland; 2 Department of Genetics, Faculty of Biology and Environmental Protection, University of Silesia, Jagiellońska 28, 40-032 Katowice, Poland

**Keywords:** Drought, Functional annotation, Phenotyping, QTLs, *sdw1/denso* gene, Spring barley

## Abstract

High-yielding capacity of the modern barley varieties is mostly dependent on the sources of semi-dwarfness associated with the *sdw1/denso* locus. The objective of the study was to identify quantitative trait loci (QTLs) associated with the plant height and yield potential of barley recombinant inbred lines (RILs) grown under various soil moisture regimes. The plant material was developed from a hybrid between the Maresi (European cv.) and CamB (Syrian cv.). A total of 103 QTLs affecting analysed traits were detected and 36 of them showed stable effects over environments. In total, ten QTLs were found to be significant only under water shortage conditions. Nine QTLs affecting the length of main stem were detected on 2H-6H chromosomes. In four of the detected QTLs, alleles contributed by Maresi had negative effects on that trait, the most significant being the QLSt-3H.1-1 in the 3H.1 linkage group. The close linkage between QTLs identified around the *sdw1/denso* locus, with positive alleles contributed by Maresi, indicates that the semi-dwarf cv. Maresi could serve as a donor of favourable traits resulting in grain yield improvement, also under water scarcity. Molecular analyses revealed that the Syrian cv. also contributed alleles which increased the yield potential. Available barley resources of genomic annotations were employed to the biological interpretation of detected QTLs. This approach revealed 26 over-represented Gene Ontology terms. In the projected support intervals of QGWS_l_-5H.3-2 and QLSt-5H.3 on the chromosome 5H, four genes annotated to ‘response to stress’ were found. It suggests that these QTL-regions may be involved in a response of plant to a wide range of environmental disturbances.

**Electronic supplementary material:**

The online version of this article (doi:10.1007/s13353-016-0358-1) contains supplementary material, which is available to authorized users.

## Introduction

Barley (*Hordeum vulgare* L.) is one of the first crops to be domesticated and currently is the fourth most important cereal, covering approximately 10 % of the worldwide acreage of cereals (FAOSTAT 2013).

The spring barley is characterized by a wide range of adaptation to various conditions—it is cultivated in temperate climate with sufficient rainfall as well as in marginal environments with intensified occurrence of drought (Ceccarelli 1994). Although it is better adapted to variable water availabilities than other cereals, drought is one of the major environmental stresses constraining its growth and productivity (López–Castañeda and Richards 1994; Yordanov et al. 2000; Araus et al. 2002; Forster et al. 2004; Ozturk and Aydin 2004; Szira et al. 2008; Anjum et al. 2011; Rahdari and Hoseini 2012). Response to drought stress is manifested at the whole-plant level including numerous morphological, physiological and biochemical changes (Blum 1996; Anjum et al. 2011).

Different plant strategies are known to cope with the limited water supply (Levitt 1972; Ludlow 1989); among them the strategy to drought escape appears to be essential. Drought escape is a life cycle adjustment by a rapid growth and early maturation to avoid the late season stress (Blum 2005; Franks et al. 2007). This strategy mainly relates to plants grown in arid or semi-arid areas with regular water deficits. Nevertheless, due to an acceleration of the grain filling and ripening, a significant yield reductions may occur (Desclaux and Roumet 1996).

One of the features that may affect plants response to the water shortage is their height. Among numerous genes controlling the plant height in barley, the semi-dwarfing *sdw1/denso* gene is one of the most important and it has been incorporated into many modern, high-yielding cultivars (Hedden and Kamiya 1997; Kuczyńska et al. 2013). It was mapped on the long arm of chromosome 3H (Barua et al. 1993) and several molecular markers were reported to be co-located with the *sdw1/denso* region, mostly the microsatellite Bmag0013 (e.g. Baum et al. 2003; Kuczyńska et al. 2014). Recently, SNP 6716–823 (11_10867) (Malosetti et al. 2011) and SNP 5260–462 (11_10754) (Pasam et al. 2012) have been suggested to be linked to the *sdw1/denso* locus. These SNP markers are mapped at a distance less than 1 cM from each other (Close et al. 2009). The *Hv20ox2* gene has been reported as the functional gene of the *sdw1/denso* locus (Jia et al. 2015). Plants bearing the *sdw1/denso* gene are characterized by prostrate growth habits at the juvenile stage. Such attributes results in a dense ground cover around plants and in the reduction of water evaporation from soil as a consequence (Baum et al. 2003). Thus, the prostrate barleys may exhibit higher yields under drought conditions (Ceccarelli et al. 1991).

Recently, considerable progress has been made in understanding the *sdw1/denso* gene structure and its functions (Jia et al. 2009, 2011, 2015; Kuczyńska and Wyka 2011; Kuczyńska et al. 2012, 2014), but knowledge about effects of this gene on agronomic traits under water shortages is still limited. Nevertheless, some data suggest its favourable effects on yield in dry environments. Diab et al. (2004) detected QTLs that controlled osmotic potential and Talamè et al. (2004) found QTLs that determined a prostrate/erect growth habit—both co-segregating with microsatellite marker Bmag0013. Moreover, Chloupek et al. (2006) identified a QTL associated with the root development at the *sdw1/denso* region and assumed that semi-dwarf plants formed more extensive root systems.

The aim of this study was to detect QTLs affecting important agronomic traits under temporary water shortages in a recombinant inbred line population (RIL) developed from a cross between European and Syrian genotypes. Plant height was considered with a special attention paid to the effects of the *sdw1/denso* locus on the plant growth and productivity under drought.

## Materials and methods

### Materials

Spring barley (*Hordeum vulgare* L.) population (hereafter named as MCam) of recombinant inbred lines (RILs) derived from the cross Maresi × Cam/B1/CI08887//CI05761 (hereafter referred as CamB) was used in our studies. RILs were developed by the single-seed descent (SSD) technique till F_8_ generation. The plant material was described in detail by Mikołajczak et al. (2016). About 150 RILs were developed and out of them 100 were randomly chosen for experiments in which their response to temporal drought stresses was assessed.

A detailed protocol of RILs genotyping by simple sequence repeat (SSR) and single nucleotide polymorphism (SNP) markers as well as a genetic map construction based on the MCam population and two other bi-parental RIL populations was described by Mikołajczak et al. (2016).

### Temporary water scarcity experiments

Experiments were conducted in 2011–2013 under greenhouse conditions. In each year, seeds of 100 RILs and parental genotypes were sown in the first week of April. Before sowing, seeds were treated with ‘Funaben’ (Organika-Azot, Jaworzno, Poland) at the rate of 1.5 g kg^−1^ to control seed-borne diseases. Double-walled Kick-Brauckman’s pots of 10 dm^3^ capacity were used for experiments.

Each pot was filled with 9 kg of loamy soil taken from experimental fields and mixed with sand in weight proportion 7:2. Chemical and physical properties of the prepared soil mixture were determined by the Institute of Soil Science and Plant Cultivation – State Research Institute (Puławy, Poland). On the basis of the chemical analysis, doses of macro- and micro-nutrients per each pot were established according to the Fertilization Recommendation (1990) and Jadczyszyn et al. (2008). Before sowing, macro- and micro-nutrients were applied into the each pot in the following rates: 0.9 g P as KH_2_PO_4_; 1.62 g K as KH_2_PO_4_ and K_2_SO_4_; 0.6 g Mg as MgSO_4_ × 7H_2_O, 5 mg H_3_BO_3_; 2 mg CuSO_4_; 10 mg MnSO_4_ × 4H_2_O and 50 mg Fe(C_6_H_5_O_7_) × 3H_2_O. Nitrogen was applied in two doses, 1.2 g each as NH_4_NO_3_: just after sowing and at the end of tillering stage.

**Fig. 1:**
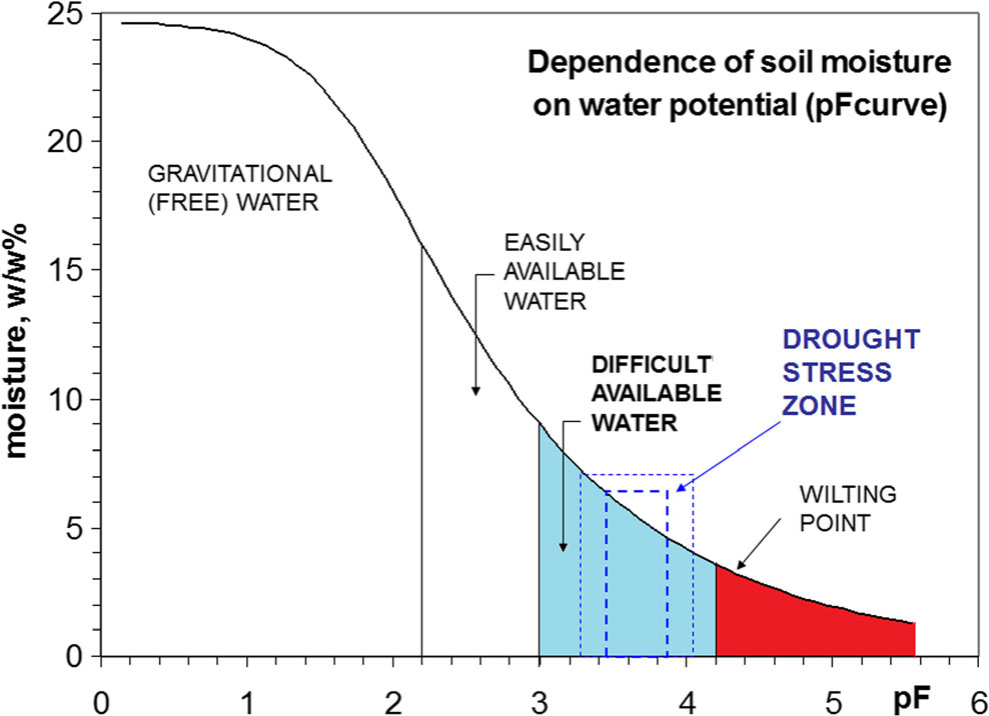
Water retention curve pF

The prepared soil was characterized physically by water retention curve pF (Fig. 1), as developed by the Institute of Agrophysics of the Polish Academy of Sciences (Lublin, Poland). The pF value is defined as a logarithm of the pressure *p* (expressed in centimeters of water head) necessary for removal of water from soil capillaries. Easily available water for plants situates between pF 2.2 and 3.0; at pF 3.0-4.2 water is less available, while at pF > 4.2 water is unavailable being a permanent wilting point.

Three water regimes (treatments) were applied in the experiment:

- C – control (well-watered) conditions: pF 2.2-3.0 (easily available water) during the whole plant vegetation;
- T-I – 10-day drought stress beginning at the 3-leaf stage (13 BBCH scale), pF 3.4-3.6;
- T-II – 14-day drought stress beginning at the flag leaf stage (37 BBCH scale), pF 3.4-3.6.

During drought stress, soil moisture was maintained in the interval of difficult available water, but always this was above the permanent wilting point. In treatments T-I and T-II, i.e. in periods outside the drought, the soil moisture was maintained at the same level as in the control.

Soil moisture in each pot was controlled gravimetrically by weighing and, additionally, volumetrically (if necessary) by an use of the FOM/mts, i.e. a time domain reflectometry based microprocessor controlled device (constructed by the Institute of Agrophysics, Lublin, Poland). In the greenhouse the air was mixed by fans, which provided uniform temperature and air circulation.

Experiments were conducted as follows: 2011 – control (C_11) and treatment I (T-I_11), 2012 – control (C_12) and treatment II (T-II_12), 2013 – control (C_13), treatment I (T-I_13) and treatment II (T-II_13).

In each pot, 20 seeds were sown and after emergence the number of plants in pots was reduced to ten. In 2011, each genotype in each treatment was grown in three pot-replications, whereas in 2012 and 2013 each genotype in each treatment was sown in two pot-replications. Experiments were carried out in a completely randomized block designs. Experiments in the consecutive years were considered as carried out in separate environments (planned treatments across three years = seven environments) and not as repetitions.

### Phenotyping

All plants from each pot were hand-harvested and the following traits were observed and expressed as a mean of ten plants: length of the main stem (LSt); i.e. length of the stem from ground level to the end of spike, without awns; number of productive tillers per plant (NPT); i.e. number of tillers with spikes containing grains; length of main spike (LS_m_); i.e. length of mature spike from the main stem measured from the spike base to its top, without awns; number of grains per main spike (NGS_m_); grain weight per main spike (GWS_m_); length of lateral spike (LS_l_); i.e. length of a mature spike from the lateral stem measured from the spike base to the top, without awns; number of grains per lateral spike (NGS_l_); grain weight per lateral spike (GWS_l_); 1000-grain weight (TGW); as calculated for grains collected from all plants per pot; grain weight per plant (GWP); weight of grains from one plant. During the vegetation, the date of heading (HD) was noted (51 BBCH scale) and expressed as the number of days from sowing.

### Statistical analyses

Observations of phenotypic traits were subjected to analysis of variance in the mixed model with fixed effects of year, treatment, and of year × treatment interaction, and with random effects of RILs and interaction of RILs with year and treatments. Residual maximum likelihood (REML) algorithm was used to estimate variance components for random effects and *F*-statistic was computed to assess the significance of fixed effects. Broad-sense heritabilities were computed from appropriate variance components. Pearson correlations were used to evaluate associations between the length of main stem and other characteristics. All these analyses were performed using Genstat 16-package (VSN Int. 2013).

QTL localization was performed in the consensus linkage map (Mikołajczak et al. 2016) using the method described by Malosetti et al. (2013) implemented in Genstat 16 (VSN Int. 2013). Only lines with less than 20 % of missing genotype data were included in this analysis. After selecting the best covariance structure to model genetic correlations among environments, interval mapping was done with the step size of 2 cM by first selecting candidate QTL and then using them iteratively as cofactors until the list of QTL was not changed. The threshold for − log_10_(*P*-value) statistic, hereafter referred as LogP, was computed by the method of Li and Ji (2005) to ensure that the genome-wide error rate was smaller than *P* = 0.01. The windows for not selecting two close QTLs and for exclusion of cofactors were set at 10 and 30 cM, respectively. Selection of the set of QTL effects in the final model was done at *P* < 0.05; the *P*-values for the Wald statistic were computed as the mean from the values obtained by adding and dropping the QTL main and interaction effects in the model. For each QTL the two LOD QTL support interval was computed according to Xu (2010); mean length of all intervals, *m*, was further used as a parameter in QTL annotation.

### QTL annotation

QTL annotation was achieved by mapping all SNP sequences taken from Close et al. (2009) (supplementary material file BOPA1 SNP 1471-2164-10-582-S19.xls) to barley genome space in EnsemblPlants (ver. 082214v1, reference repeat masked sequence Hordeum_vulgare.082214v1.28.dna_rm.toplevel.fa, NCBI Blast for Windows with maximum EValue = 1e-060, minimum 95 % identity of the SNP sequence). For each QTL, the interval of length *m* around the closest SNP was projected onto the genomic sequence with the use of SNPs corresponding to the interval boundaries; all genes located in projected intervals were listed and annotated using Gene Ontology (GO) terms. The homogeneity of GO term distribution in various QTL regions was checked by the χ2 test; the GO terms over-represented in particular regions were identified by significant positive normalized deviations from homogeneity.

## Results

### Phenotypic variation of RIL population

Large differences in the analysed traits were found between parents as well as within the RIL population. Mean values and standards errors of observed traits are presented in electronic supplementary material (ESM 1).

In general, cv. Maresi was characterized by higher values of studied traits, excluding LSt, than CamB under both well-watered and stress conditions. The main trait which differentiated the parental genotypes was GWP; grain weight per plant for Maresi was from 22 to 48 % higher than that for CamB. The Syrian parent was found to be definitely earlier than Maresi, and HD for CamB was observed to be 10 to 17 days earlier than in Maresi.

RILs varied in the studied traits and their extremes exceeded the range pointed by the better or the worse scoring parent, therefore a considerable transgressive segregation was observed (ESM 1). In general, the studied traits were more affected by T-II than T-I. The reduction of LSt as a result of water shortage was about 10 % in T-I_11 and 22 % in T-II_13. Among observed traits, the GWP was the strongest limited by the water shortage. Decrease in GWP under T-I and T-II was about 30 % in years 2011 and 2012 and above 50 % in T-II_13in years 2011 and 2012, and above 50 % in T-II_13.

Coefficients of variation (CVs) for the analysed traits varied in different environments. CVs for GWP, LS_m_ and NGS_m_ ranged from *ca*. 13 to 20 % and their slightly higher values were found in both stress conditions (T-I, T-II) as compared to the control (C) (ESM 1). Otherwise, the range of variability in LSt was greater in C than in T-I or T-II; however, the CVs were relatively low in all cases (max. approx. 13 %). Among the analysed traits, the lowest CV was found for HD – ≈ 5 % in T-II_12 and it did not exceed 12 % in other environments. Similarly, low CVs were obtained for TGW (about 6-13 %). The rest of the traits were characterized by CVs ranging from several percent to *ca*. 20-24 %, with the highest CV found for GWS_l_ (≈24 % in T-II_12).

Results of analysis of variance showed significant effects of year, treatment and their interaction for all analysed traits at least at *P* = 0.001. Also, the variance components for RILs were significant for observed characteristics. For each trait, RIL × year interaction was important (except for NPT), whereas RIL × treatment interaction was found to be significant only for NPT, LS_m_, GWS_m_, NGS_l_, and HD. Variance components of the RIL × year × treatment interaction were significant for LSt, GWS_m_, GWS_l_, TGW and HD (Table 1). Note, that variance components calculated for GWS_m_ and HD were significant in all cases.

**Table 1:** Variance components and standard errors (s.e.) estimated for phenotypic traits observed in barley RIL population, broad-sense heritabilitiesies heritability (h^2^_bs_) calculated across different treatments

Trait	Variance component, standard error (s.e.)								Broad sense heritability (h^2^_bs_)		
	RIL	s.e.	RIL × year	s.e.	RIL × treatment	s.e.	RIL × year × treatment	s.e.	T-I	T-II	C
LSt	31.73*	5.34	8.51*	1.60	2.05	0.97	6.41*	1.34	83.58	72.44	84.84
NPT	0.13*	0.03	0.05	0.02	0.08*	0.02	0.04	0.02	40.73	58.49	83.53
LS_m_	0.737*	0.115	0.106*	0.016	0.062*	0.012	0.011	0.012	90.96	94.01	91.69
NGS_m_	6.96*	1.06	0.49*	0.13	0.24	0.11	0.43	0.15	89.86	81.98	95.01
GWS_m_	0.013*	0.002	0.002*	0.000	0.001*	0.000	0.002*	0.000	88.06	92.29	88.84
LS_l_	0.533*	0.083	0.093*	0.015	0.006	0.008	0.022	0.012	90.73	-	87.99
NGS_l_	3.84*	0.64	0.84*	0.17	0.46*	0.13	0.16	0.17	81.97	64.00	90.89
GWS_l_	0.005*	0.001	0.002*	0.000	0.001	0.000	0.002*	0.000	54.94	58.45	82.04
TGW	5.59*	1.17	4.81*	0.79	−0.24	0.37	2.57*	0.64	48.92	43.81	59.59
GWP	0.032*	0.008	0.037*	0.006	0.005	0.004	0.008	0.005	36.06	76.19	54.98
HD	18.19*	2.86	3.39*	0.43	1.41*	0.25	1.55*	0.18	88.41	92.68	94.11

*statistical significance of variance components was defined by comparing their values with standard error (component was significant if its value was greater than three times of standard error value); missing data of h^2^_bs_ – negative value of heritability

Broad-sense heritabilities (h^2^_bs_) for the observed traits were diversified (Table 1). The h^2^_bs_ values for LSt were high and ranged from 72 to 85 %. The consistent and substantial heritability (more than 90 %) was found for LS_m_ across all experiments. The lowest h^2^_bs_ was identified for GWP in well-watered conditions (≈55 %) and in the treatment T-I (≈36 %), and for GWS_l_ in the treatment T-II (≈58 %).

Correlation coefficients between LSt and other traits indicate that LSt was positively associated with the majority of discussed traits, with the exception for NPT for which in all significant cases the negative correlations were revealed. The correlations between LSt and HD were less consistent, i.e. negative relations in C_11 and T-II_12 and positive associations in T-I_13 and T-II_13 were found (Table 2).

**Table 2:** Pearson correlation coefficients between length of the main stem and other phenotypic traits in the barley RIL population under different environments; significance at *P* < 0.001

Trait	Length of main stem (LSt)						
	T-I_11	C_11	T-II_12	C_12	T-I_13	T-II_13	C_13
NPT	n.s.	n.s.	−0.35	−0.51	−0.54	n.s.	−0.50
LS_m_	0.31	n.s.	0.23	0.32	0.64	0.56	0.54
NGS_m_	0.47	n.s.	n.s.	n.s.	0.50	0.59	0.34
GWS_m_	0.60	0.39	n.s.	n.s.	0.54	0.59	0.44
LS_l_	0.31	n.s.	n.s.	0.28	0.57	0.58	0.40
NGS_l_	0.49	n.s.	n.s.	n.s.	0.24	0.24	n.s.
GWS_l_	0.58	0.42	n.s.	n.s.	0.32	0.30	n.s.
TGW	0.29	0.37	0.35	n.s.	0.33	0.28	0.26
GWP	0.49	0.36	n.s.	−0.29	n.s.	0.36	n.s.
HD	n.s.	−0.43	−0.24	n.s.	0.33	0.43	n.s.

n.s. – not significant

### QTL analysis

A total of 103 QTLs were mapped for the observed traits onto the seven chromosomes (see electronic supplementary material ESM 2). The most QTLs were positioned in the linkage group 2H (28), while no any QTL was found in 1H.2 and 5H.2. The largest number of QTLs was detected for GWS_m_ (13) and the smallest for HD (4). The significant QTL × E interaction was noted for 65 % of all the detected QTLs. Five QTLs showed significant effects exclusively under the well-watered conditions, and ten—only under water shortage conditions.

### Length of the main stem (LSt)

**Fig. 2:**
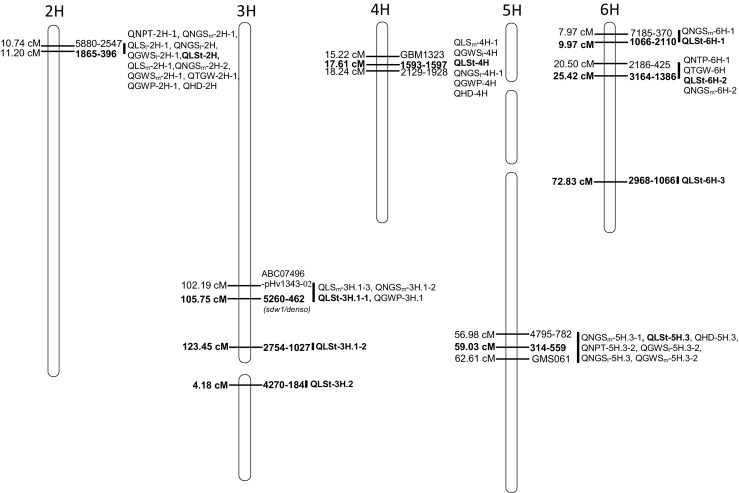
The co-localization of the QTLs for the length of main stem (bolded) and other traits (±5 cM); names of the closest linked markers and QTL symbol on the right side and their position in cM – left side; no QTLs for LSt were detected on 1H and 7H chromosomes; diagram was designed on the base of the genetic linkage map constructed by Mikołajczak et al. (2016)

Length of the main stem was affected by nine QTLs detected in the 2H, 3H.1, 3H.2, 4H, 5H.3 and 6H linkage groups. Five of them exhibited stable effects (QLSt-3H.2, QLSt-4H, QLSt-5H.3, QLSt-6H-1, QLSt-6H-2). In four of detected QTLs alleles contributed by cv. Maresi reduced the trait, the most significant being the QLSt-3H.1-1 linked to SNP 5260–462 in the 3H.1 linkage group (105.75 cM). This QTL showed an interaction with environments, and the QTL effects were higher in well-watered than in water shortage treatments. Two other QTLs with positive effects of Maresi alleles were QLSt-2H (at SNP 1865–396) and QLSt-5H.3 (at SNP 314–559, stable over environments). Position of all QTLs for LSt and their co-localization with studied traits is presented in Fig. 2 and in EMS 2.

### Number of productive tillers per plant (NPT)

Twelve QTLs were found for NPT, four of them in the linkage group 2H. Five QTLs (QNPT-2H-2, QNPT-5H.1, QNPT-5H.3-1, QNPT-6H-1, QNPT-7H.2) were stable across the environments. The most important QTL was mapped in 2H at SNP 5880–2547 in which CamB alleles increased the trait in well-watered and T-I treatments and decreased in T-II. Effects of two QTLs — QNPT-2H-3 and QNPT-2H-4 were significant only under the water shortage (EMS 2).

### Main spike characteristics

Twelve QTLs affecting LS_m_ were distributed onto all chromosomes, excluding 6H. Five of them (QLS_m_-1H.1, QLS_m_-2H-3, QLS_m_-2H-4, QLS_m_-4H-1, QLS_m_-7H.2) had effects independent on the environment. The most significant, QLS_m_-2H-1, was positioned in 2H at SNP 1865–396; its additive effect was negative and dependent on the environment, with a large portion of variance explained (above 21 %). Two QTLs (QLS_m_-2H-2, QLS_m_-3H.1-3) were significant only in experiments with limited water in early stages of development (T-I_11, T-I_13).

Twelve QTLs were identified for NGS_m_. Five of them (QNGS_m_-2H-2, QNGS_m_-2H-3, QNGS_m_-3H.1-1, QNGS_m_-5H.1, QNGS_m_-7H.2), had stable effects. All QTLs detected in 2H as well as one in 3H.1, one in 5H.3, two in 6H and one in 7H.2 had positive effects associated with Maresi allele. In the rest of the loci, CamB allele increased the trait. The QNGS_m_-2H-1, i.e. the most important QTL (at SNP 5880–2547), was significant only in C_11 and C_12. There were three QTLs influencing NGS_m_ only in treatment T-II. For two of them, mapped in 3H.1 and 6H (QNGS_m_-3H.1-2, NGS_m_-6H-2), alleles increasing the number of grains in main spike originated from Maresi (EMS 2).

Among 13 QTLs identified for GWS_m_, four (QGWS_m_-2H-2, QGWS_m_-2H-3, QGWS_m_-3H.1-1, QGWS_m_-5H.3-3) were independent on environments. The main QTLs were located in 2H (at SNP 1865–396), and 5H.3 (at SNP 603–72); the allelic effects of these two QTLs were of opposite sign (EMS 2).

### Lateral spike characteristics

LS_l_ was determined by ten QTLs positioned in 2H, 3H.1, 4H, 5H.3, 7H.2 linkage groups and four of them (QLS_l_-2H-2, QLS_l_-2H-3, QLS_l_-3H.1-1, QLS_l_-7H.2) showed stable effects. Two major QTLs (LogP >13) with opposite effects of Maresi alleles were found: QLS_l_-2H-1 enhancing the trait, and QLS_l_-5H.3-3 reducing the trait. (EMS 2).

Excluding for 1H, nine QTLs located in residual chromosomes were found for NGS_l_ and three of them (QNGS_l_-2H, QNGS_l_-6H-1, at QNGS_l_-7H.2) were independent on the environment. Maresi allele increased the trait at all three of these stable loci (EMS 2).

GWS_l_ was determined by seven QTLs located in 1H.1, 2H, 4H, 5H.3, 7H.2 linkage groups. Among them QGWS_l_-7H.2 exhibited stable effects across the environments and QGWS_l_-2H-2 had significant effect only under water deficit (T-II_13). The major QTL was QGWS_l_-2H-1 located at SNP 5880–2547. In the QGWS_l_-2H-1 and QGWS_l_-2H-2 Maresi alleles increased the trait (EMS 2).

### Other traits

Six QTLs identified in 2H, 4H, 5H.3 and 6H linkage groups affected TGW. Two of them (QTGW-2H-2, QTGW-6H) were independent on the environment, with CamB alleles reducing the trait. Positive effects of CamB allele on TGW were revealed in QTGW-2H-1 localized at SNP 1865–396 but they were dependent on environment (EMS2).

Nine QTLs were identified for GWP in 2H, 3H.1, 4H, 5H.3, 6H and 7H.2. Two QTLs (QGWP-3H.1, QGWP-4H) exhibited stable effects across the environments, but they were of opposite signs. The main QTL was QGWP-2H-1 (at SNP 1865–396), with Maresi allele increasing the trait. QGWP-2H-3 and QGWP-6H were identified to be significant only under water shortage applied at T-II (EMS 2).

Four QTLs were found for HD located in 2H, 4H, 5H.3 and 7H.2 linkage groups and all of them showed interaction with environment. The strongest effect was recorded for the QHD-2H positioned at SNP 1865–396, with delayed heading determined by Maresi allele. This QTL explained a large percentage of phenotypic variation (above 93 %) (EMS 2).

### QTL annotation

The estimated mean length of two LOD QTL support intervals, *m*, was equal to 5.4 cM. The length of projections of QTL support intervals onto the genomic sequence varied from 128 kbp to 337 Mbp. For interpretation, we used only annotations of genes contained in the 80 intervals (out of 103) shorter than 20 Mbp since very long intervals result from a serious distortions of the co-linearity of SNPs in the linkage map and in the genomic sequence. The proportions of genes classified according to GO-terms describing biological processes were different among the QTLs-regions (χ2 test, P < 0.001); the 26 GO terms over-represented in at least one QTL region, together with the total number of genes and lists of genes annotated with these terms in particular regions, are listed in Table 3. The number of genes located in the considered intervals varied from seven to 228. The number of genes classified according to over-represented GO-terms ranged from one to nine. One out of these genes, surrounding the QGWS_l_-5H.3-2, was found to have defined a transcript name—MLOC_51191 (HsfA2a). The GO-term ‘protein ubiquitination’ was the most numerously represented by genes which were identified around the QLS_l_-2H-3.

**Table 3:** GO biological process terms over-represented in the annotation of genes occurring in the regions of QTLs

GO biological process term	QTL symbol	Total number of genes in the QTL region	Number of genes annotated with the term	List of genes
carbohydrate metabolic process	QGWS_l_-4H	7	1	MLOC_69905
defense response	QLS_m_-3H.1-1	82	2	MLOC_3038, MLOC_50823
	QLSt-6H-2	41	2	MLOC_38183, MLOC_76360
	QNGS_m_-7H.2	102	5	MLOC_52432, MLOC_14659, MLOC_6883, MLOC_31061, MLOC_62757
	QNPT-5H.1	36	1	MLOC_67608
fatty acid biosynthetic process	QGWP-4H	7	1	MLOC_79574
	QLS_m_-4H-1	35	2	MLOC_55029, MLOC_54031
lipid metabolic process	QLS_l_-7H.2	146	5	MLOC_58861, MLOC_57679, MLOC_81843, MLOC_65304, MLOC_4594
	QNPT-6H-1	103	2	MLOC_35847, MLOC_19178
lipid transport	QLS_m_-2H-3	150	3	MLOC_14732, MLOC_9887, MLOC_59422
metabolic process	QLSt-2H	47	4	MLOC_44360, MLOC_12202, MLOC_51066, MLOC_25950
multicellular organismal development	QNGS_m_-3H.1-1	161	6	MLOC_37082, MLOC_63238, MLOC_71688, MLOC_50033, MLOC_56271, MLOC_81554
	QNPT-3H.1-1	77	4	MLOC_68550, MLOC_68553, MLOC_64722, MLOC_3103
negative regulation of catalytic activity	QLS_l_-3H.1-2	82	3	MLOC_72017, MLOC_52861, MLOC_16041
obsolete ATP catabolic process	QLS_l_-7H.2	146	5	MLOC_2098, MLOC_76366, MLOC_12388, MLOC_44081, MLOC_9846
phosphorylation	QHD-4H	100	6	MLOC_2842, MLOC_37279, MLOC_10839, MLOC_61764, MLOC_58063, MLOC_53722
	QLS_l_-3H.1-1	10	2	MLOC_55753, MLOC_55752
protein ubiquitination	QLS_l_-2H-3	228	9	MLOC_40031, MLOC_54978, MLOC_679, MLOC_60024, MLOC_8581, MLOC_81408, MLOC_63051, MLOC_39480, MLOC_63511
regulation of signal transduction	QLS_l_-3H.1-1	10	4	MLOC_55753, MLOC_55752, MLOC_36868, MLOC_36867
response to auxin	QGWP-5H.3	159	5	MLOC_58506, MLOC_58508, MLOC_58507, MLOC_65368, MLOC_65978
	QLS_l_-2H-3	228	7	MLOC_62887, MLOC_8923, MLOC_61988, MLOC_22418, MLOC_14016, MLOC_5261, MLOC_55346
response to freezing	QNPT-2H-1	42	1	MLOC_50785
response to stress	QGWS_l_-5H.3-2	159	3	MLOC_51191 (HsfA2a), MLOC_59425, MLOC_14293
	QLSt-5H.3	54	1	MLOC_59425
ubiquitin-dependent protein catabolic process	QNGS_m_-3H.1-1	161	6	MLOC_37082, MLOC_63238, MLOC_71688, MLOC_50033, MLOC_56271, MLOC_81554
	QNPT-3H.1-1	77	4	MLOC_68550, MLOC_68553, MLOC_64722, MLOC_3103

## Discussion

In the present studies, population of RILs derived from the cross between European cultivar Maresi and Syrian breeding line CamB was examined in experiments conducted under various water regimes. Plant height can be considered as a good indicator of plant reaction to drought since a growth inhibition is one of the first signs observed during the water deficit. The loss of turgor causes a disruption in the water transport in the plant that results in limitations in the division and elongation of cells (Nonami 1998). Plant height is negatively correlated with grain weight per plant under water shortage, particularly when it occurs in a later stage of the plant growth (Simane et al. 1993; van Ginkel et al. 1998). In the present studies, phenotypic correlations between the stem length and other traits were generally positive, except for the number of productive tillers per plant, for which correlations were negative in all experiments. The associations between stem length and days to heading appeared to be not consistent; those were negative or positive depending on environments.

### Effects of water scarcity

In the present study, a delay in heading was recorded in experiments where drought was imposed at the later stage (T-II), whereas water shortage applied in the seedling stage did not influence the heading time. Moreover, variation coefficients for days to heading were lower in T-II than in other treatments. This may indicate that water deficit at the flag leaf stage resulted in a decrease of differences in the heading time between the lines within the population. Some authors also reported that water stress causes delays in the developmental phases of cereals (Blum 1996; Winkel et al. 1997). A delay in heading under water deficit can be caused by increased biosynthesis of abscisic acid (ABA) as a plant response to stress. Such action of ABA has been shown, e.g. by Wang et al. (2013) in *Arabidopsis thaliana*. However, observations of the effect of drought on vegetative growth duration are not consistent. Some authors reported that water deficit decreases the growth duration due to the accelerated transition of plants from vegetative to generative phase, which can result in a large reduction of yield (Desclaux and Roumet 1996; McMaster and Wilhelm 2003). Cattivelli et al. (2008) observed acceleration of flowering under water stress both in wheat and barley. According to Angus and Moncur (1977) as well as Dwyer and Stewart (1987), moderate drought accelerates flowering, while deeper stress causes its delay. Discrepancies in reports may be due to the different intensities and duration of stress as well as to the various set of examined genotypes.

Water deficit applied in the present studies reduced number of developed grains in the main and lateral spikes as well as their weight, which resulted in the decrease of grain weight per plant, although the number of productive tillers increased. Lower number of grains formed in spikes may be due to a lower pollen viability during water stress. Water shortage during vegetative stages can limit the tiller development. However, after the restoration of watering, plants are capable of accelerated production of new tillers in order to compensate for a loss in biomass caused by stress, but newly formed tillers usually generate a lower yield. The negative effects of drought on grain yield were confirmed by numerous reports, which indicated that water deficit occurring at tillering, heading and grain filling stages caused the greatest loss in yield (Sakamoto and Matsuoka 2004; Blum 2005; Khomari et al. 2008; Mäkelä and Muurinen 2011).

Reduction in stem length caused by water deficit decreases the distance of water transport in the plant. According to Kuczyńska and Wyka (2011), semi-dwarf barley plants compared to tall plants are characterized by a reduced leaf length and surface of leaf blades and by a smaller diameter of xylem vessels. Such changes in anatomy of leaves can influence the water transport which will be more efficient and transpiration will be reduced as a consequence. In dry conditions, such phenomena may increase the survival of plants. Yin et al. (1999) reported an increased biomass in semi-dwarf plants and Farooq et al. (2010) found a reduction of the number of leaves by drought through the acceleration of their aging. Thus, an improved biomass of leaves and extensive root system, as observed by Chloupek et al. (2006) in semi-dwarf barley plants, can favourably affect the yield potential of plants under drought conditions.

### QTLs for the plant height

In barley, plant height is conditioned by several dwarfing and semi-dwarfing genes (e.g. Abeledo et al. 2002; Maluszyński and Szarejko 2005; Araus et al. 2008; Kuczyńska et al. 2013). In the present studies the most significant QTL for length of main stem was found in 2H in the region of SNP 1865–396. Close to it, QTLs for all other traits were also located. In most cases these QTLs exhibited the strongest effects on traits, so this interval seems to be important for the analysis of genetic determination of the observed traits. Several reports also considered this 2H region as a hot-spot (e.g. Wang et al. 2010; Mansour et al. 2014). However, we observed that in this region, represented in the integrated linkage map by six markers (5880–2547, 1865–396, GBM1214, 7766–492, 8787–1459, 7747–1056), segregation existed in five markers (with the exception of GBM1214), but the marker data were almost co-linear, with just one line showing recombination between 1865–396 and 7766–492. We conclude that the region between 10 and 13 cM on 2H is not well described by markers in the studied population, which—especially—does not allow for discrimination between tight linkage and pleiotropy. Because of the segregation problems, we decided to postpone the interpretation of the said region until better SNP (iSelect) data is available.

The second most significant QTL for length of main stem was positioned in 3H.1 linkage group at SNP 5260–462 (11_10754). Additive effect of that QTL was relatively lower in experiments with water shortages than in the well-watered conditions. Similar results were reported by Baum et al. (2003); among seven QTLs identified by those authors for plant height the greatest influence on this trait was found for QTL localized at the *sdw1/denso* locus and its effect was significant in experiments conducted in dry environments. Malosetti et al. (2011) localized QTL for plant height at the same region but linked to SNP 6716–823 (11_10867) in the distance of 1 cM from SNP 5260–462. Authors claimed that the *sdw1/denso* locus is positioned there, what was also revealed earlier, among others, by Barua et al. (1993) and Hellewell et al. (2000). At that locus, Jia et al. (2009, 2011) identified the *Hv20ox2* gene encoding gibberellin 20-oxidase enzyme; the authors observed that semi-dwarf plants exhibit a reduced expression of this gene, and at the same time an increased number of tillers and higher grain yield. We checked that the sequence of the A/G SNP found by Jia et al. (2009) in the intron of the *Hv20ox2* gene maps in Ensembl Plants to MLOC_56462 (in its intron, at position 3:509044937–509045076, %ID = 99.2, E-value = 2.1E-62, with a corresponding SNP), that has been recently prioritized as the barley GA-20 rice and wheat ortholog (Pearce et al. 2015). Because SNP 6716–823 maps in Ensembl at the distance of about 134 kb from MLOC_56462 (in the same contig), and SNP 5260–462 at the distance of more than 1 Mbp (in the nieghbouring contig), the plant height QTL position found by Malosetti et al. (2011) seems to be closer to the candidate *GA-20* gene than our QTL. However, Pasam et al. (2012) found the QTL for plant height linked to the same SNP on 3H chromosome as in our studies. Association between a reduced plant height (caused by *sdw1/denso*) and other traits, including grain yield, was recorded in numerous studies, although the results are not consistent. For example, Thomas et al. (1991) and Hellewell et al. (2000) observed decreased yield, whereas Yin et al. (1999) and Jia et al. (2011) noticed increased yields of semi-dwarf plants. In the present studies, QTL for yield identified on 3H (QGWP-3H.1) was linked to the same SNP (5260–462) as QTL for length of main stem (QLSt-3H.1-1). Moreover, QGWP-3H.1 had stable effects across environments and Maresi allele increased grain yield. Jia et al. (2011) found QTL for grain yield as co-localizing with the *Hv20ox2* gene and increased yield was caused by an allele from the cv. Baudin carrying *sdw1/denso*. Authors concluded that decreased expression of the *Hv20ox2* gene reduces production of gibberellins, what limits the growth of apical meristem and at the same time promote the formation of more tillers per plant. In the present studies QTL for number of tillers, QNPT-3H.1-2, found on 3H, was linked to SNP ABC06381-1-5-73 (11_11141) at 114.48 cM, but with the shift of 5.38. Besides QTL for grain weight per plant in that region on 3HL QTLs for length of main spike (QLS_m_-3H.1-3) and number of grains in main spike (QNGS_m_-3H.1-2) were identified. Increased NGS_m_ values were associated with allele from the cv. Maresi. It should be stressed that effects of QNGS_m_-3H.1-2 were significant only in T-II, i.e. during water deficit at the flag leaf stage, whereas QLS_m_-3H.1-3 only in T-I, i.e. during water stress at the seedling stage. Both QTLs were linked to the same SNP (ABC07496-pHv1343-02). Therefore, at water shortage occurring at the early stage of plant development the allele from Syrian CamB can increase spike length, while at water stress occurring at the later stages Maresi allele can contribute to a higher number of grains developed in spikes.

A QTL for length of main stem of major effect was also found in the 5H.3 linkage group (QLSt-5H.3), but in contrast to QLSt-3H.1-1, it had stable effects over environments and the allele contributed by the Syrian parent reduced the stem length. Similar results were reported by Malosetti et al. (2011), who found two major QTLs for plant height, i.e. on 3H in the *sdw1/denso* region and on 5H in the *ari-e.GP* region. Taking into account SSR and SNP markers linked to the *ari-e.GP* as well as barley maps developed by Varshney et al. (2007) and Close et al. (2009), we can conclude that this region corresponds to the linkage group 5H.2 in our map, but no QTL for stem length was localized in our studies in that group. On that chromosome, QLSt-5H.3 was found in 5H.3 linkage group at 58.01 cM and was linked to SNP 314–559 (11_20487). Around position of the QLSt-5H.3, several QTLs for yield-forming traits were mapped. In that region Rode et al. (2012) also revealed QTLs for plant height, whereas Tondelli et al. (2014) apart from the plant height found QTLs for heading, spike length and grain yield. Authors concluded that QTLs detected in this region co-localize with the vernalization response locus *Vrn-H1*. Comadran et al. (2011) reported on association between heading and SNP 11_11090. In our studies QTL for days to heading (QHD-5H.3) was linked to SNP 314–559 (11_20487) mapped at the distance 1.97 cM from SNP 11_11090.

Three QTLs for stem length were detected in our studies on 6H chromosome. Similarly, on the same chromosome Li et al. (2006) and von Korff et al. (2008) localized two QLTs, and Pasam et al. (2012)—three QTLs for plant height. Note, however, that in numerous studies no QTL for plant height was found on this chromosome (e.g. Pillen et al. 2003; Talamè et al. 2004; Malosetti et al. 2011; Rode et al. 2012; Wang et al. 2014).

### Stable and water stress specific QTL effects

In the present studies, about 1/3 (36/103) of QTLs were characterized by stable effects and they were detected for all the studied traits, except for days to heading. QTLs with stable effects detected for different traits were frequently positioned at the same region of the genome. For example, the co-localization of QTLs with such stable effects was found on 2H at 37 cM, where QTLs for length of lateral spikes (QLS_l_-2H-2) and grain weight per main spike (QGWS_m_-2H-2) were detected to be linked to SNP 2417–924 (11_10297) and in which increased trait values were contributed by CamB alleles. On that chromosome at 49.79 cM, QTLs for grain weight per main spike (QGWS_m_-2H-3) and 1000-grain weight (QTGW-2H-2) were identified, in which Maresi alleles increased the traits, as well as QTL for number of tillers (QNPT-2H-2), in which CamB allele showed stable and positive effects. Similar results were recorded by Li et al. (2006), who identified on 2H between Bmag0134 (2HL) and Bmag0518 10 QTLs responsible for spike length, number of tillers and 1000-grain weight. As a further example, in the 3H.1 linkage group between 23 and 27 cM co-localization of QTLs with positive and stable effects of CamB alleles were found for the length of main and lateral spikes (QLS_m_-3H.1-1, QLS_l_-3H.1-2), grain weight per main spike (QGWS_m_-3H.1-1) and number of grains per lateral spike (QNGS_l_-3H.1-1)—each QTL linked to SNP 3688–1291 (11_20607).

Progress in research on the formation of yield components under water scarcity can be achieved not only by the identification of QTLs of stable effects in various conditions but also by searching for regions of the genome active only under stress conditions. In the present studies, we detected ten QTLs with effects significant only under water stress in the 2H (5), 3H.1 (2), 5H.3 (1) and 6H (2) linkage groups. Three of them were associated with NGS_m_, two with NPT, LS_m_ and GWP and one with GWS_l_. Generally, among those QTLs, Maresi alleles had positive effects under water stress applied at the flag leaf stage, whereas CamB alleles showed positive effects under stress occurring at early stage of plant development. The positive impact of CamB alleles may be justified because of the fact that it has shortened vegetation period and comes from the arid region. Therefore, it is naturally adjusted to the lack of water since the early growth.

### Functional annotation of QTLs

The biological interpretation of gene sets surrounding the QTLs was patterned on Mikołajczak et al. (2016). The current resources of genomic annotations are valuable to interpret the QTLs, however we are restrained in such approach due to the limited resolution of the linkage map employed to QTLs mapping. Taking into consideration that this article discusses the impact of water shortage to yield-forming traits in barley, the GO term ‘response to stress’, over-represented in QGWS_l_-5H.3-2 and QLSt-5H.3 intervals, seems to be especially remarkable. Out of genes assigned to this biological process, the MLOC_14293 gene is the ortholog to i.a. *Aegilops tauschii* gene *F775_28135* (sequence similarity 80 %) described as ‘universal stress protein A-like protein’. Moreover, the MLOC_5191 gene was found as the only one gene with a defined transcript name (HsfA2a) that encodes heat-shock transcription factor A2. It does not only play an important role in the development of thermo-tolerance in plants but also can be a key regulator in response to several types of environmental stresses such as high salt and osmotic stress (Ogawa et al. 2007; Yokotani et al. 2008). Guo et al. (2014) performed the phylogenetic analysis of the HsfA2a amino acid from 31 plant species, including *Hordeum vulgare*, and found that this factor had the closest genetic relationships with that present in *Lycopersicon peruvianum* and *Mimulus guttatus*. Additionally, the genetic relationships of *HsfA2* in the other species having similar botanical classifications were also close. For this reason, it is used most commonly in molecular systematical analysis (Whelan et al. 2001).

## Conclusions

The close linkage between QTLs responsible for yield-forming traits, identified around the *sdw1/denso* locus with positive alleles contributed by Maresi, indicates that the semi-dwarf genotype can be a donor of favourable traits resulting in grain yield improvement, also under water scarcity. Our results indicated the region in 2H around SNP 5880–2547 (11_21015) and SNP 1865–396 (11_10180) as an acting key role in yield formation—in that region QTLs for all observed traits were detected, but interpretation of all QTL effects in this region appeared to be not possible on the basis of analysed population.

Stable and positive effects of Syrian parental genotype alleles observed for some yield-related traits, such as the number of productive tillers, length of main and lateral spikes can be one of the reasons of its adaptation to unfavourable environmental conditions, including the drought. On the other hand, unstable effects of Syrian variety alleles increasing values of some yield-forming traits during water deficit occurring in the seedling stage indicate that it can be used as a donor of genes for improved resistance of barley genotypes to drought occurring at early stages of plant development.

## Electronic supplementary material

Below is the link to the electronic supplementary material.

ESM 1 (DOCX 57 kb)

ESM 2 (XLSX 30 kb)
