# Supplementary material for: Quantitative trait loci for plant height in Maresi × CamB barley population and their associations with yield-related traits under different water regimes
Source: J Appl Genet. 2016 Jul 22;58(1):23–35. doi: 10.1007/s13353-016-0358-1 (PMC5243891; doi:10.1007/s13353-016-0358-1)
Supplement: Supplementary file 1 — (DOCX 57 kb) [file 13353_2016_358_MOESM1_ESM.docx]

EMS 1 – electronic supplementary material; Journal of Applied Genetics

Quantitative trait loci for plant height in Maresi × CamB barley population and their association with yield-related traits under different water regimes

Krzysztof Mikołajczak^a^, Anetta Kuczyńska^a*^, Paweł Krajewski^a^, Aneta Sawikowska^a^, Maria Surma^a^, Piotr Ogrodowicz^a^, Tadeusz Adamski^a^, Karolina Krystkowiak^a^, Andrzej Górny^a^, Michał Kempa^a^, Iwona Szarejko^b^, Justyna Guzy-Wróbelska^b^, Kornelia Gudyś^b^

^a^ Institute of Plant Genetics of the Polish Academy of Sciences, Strzeszyńska 34, 60-479 Poznań, Poland

^b^ Department of Genetics, Faculty of Biology and Environmental Protection, University of Silesia, Jagiellońska 28, 40-032 Katowice, Poland

*Corresponding author: tel.: (+48 61) 65 50 224; e-mail: akuc@igr.poznan.pl

Summary statistic values for traits observed for parental varieties and RILs in various experiments

| Trait  (unit) | Treatment | Parent | | | | | | | RIL population | | | | | | | | |
| --- | --- | --- | --- | --- | --- | --- | --- | --- | --- | --- | --- | --- | --- | --- | --- | --- | --- |
|  |  | Maresi | | | CamB | | | |  |  |  |  |  |  |  |  |  |
|  |  | mean | s.e. | | mean | | s.e. | | mean | | s.e. | | min. | | max. | | CV (%) |
| LSt (cm) | T-I_11 | 55.00 | 0.80 | | 54.70 | | 2.10 | | 55.45 | | 0.64 | | 40.00 | | 73.70 | | 11.48 |
|  | C_11 | 59.75 | 3.15 | | 68.10 | | 4.90 | | 61.30 | | 0.77 | | 41.90 | | 78.00 | | 12.49 |
|  | T-II_12 | 70.10 | 1.70 | | 69.20 | | 1.85 | | 66.73 | | 0.71 | | 50.55 | | 83.80 | | 10.65 |
|  | C_12 | 70.30 | 0.60 | | 73.40 | | 2.80 | | 78.40 | | 1.02 | | 53.50 | | 102.60 | | 12.97 |
|  | T-I_13 | 66.50 | 2.50 | | 68.00 | | 0.00 | | 61.48 | | 0.61 | | 49.00 | | 77.50 | | 9.86 |
|  | T-II_13 | 62.00 | 2.00 | | 61.00 | | 2.00 | | 57.18 | | 0.60 | | 44.50 | | 68.50 | | 10.45 |
|  | C_13 | 70.50 | 0.50 | | 74.50 | | 1.50 | | 72.82 | | 0.80 | | 55.50 | | 94.50 | | 11.04 |
| NPT | T-I_11 | 2.90 | 0.10 | | 3.40 | | 0.60 | | 2.90 | | 0.05 | | 1.70 | | 4.60 | | 17.32 |
|  | C_11 | 4.10 | 0.30 | | 3.90 | | 0.10 | | 3.45 | | 0.06 | | 2.10 | | 5.40 | | 18.81 |
|  | T-II_12 | 6.00 | 0.10 | | 4.50 | | 0.10 | | 5.44 | | 0.08 | | 3.20 | | 7.35 | | 14.31 |
|  | C_12 | 4.65 | 0.25 | | 4.75 | | 0.35 | | 4.15 | | 0.08 | | 2.80 | | 6.30 | | 18.67 |
|  | T-I_13 | 4.44 | 0.06 | | 3.83 | | 0.23 | | 4.29 | | 0.07 | | 2.58 | | 5.92 | | 15.89 |
|  | T-II_13 | 5.68 | 0.77 | | 3.90 | | 0.45 | | 4.08 | | 0.06 | | 2.00 | | 5.67 | | 15.31 |
|  | C_13 | 4.35 | 0.15 | | 3.95 | | 0.05 | | 4.22 | | 0.07 | | 3.00 | | 5.70 | | 15.89 |
| LS_m_ (cm) | T-I_11 | 5.85 | 0.25 | | 4.80 | | 1.00 | | 6.28 | | 0.12 | | 3.65 | | 9.05 | | 19.05 |
|  | C_11 | 7.35 | 0.25 | | 5.95 | | 0.15 | | 7.03 | | 0.10 | | 4.60 | | 9.30 | | 14.48 |
|  | T-II_12 | 6.40 | 0.20 | | 5.85 | | 0.15 | | 6.03 | | 0.09 | | 4.25 | | 8.30 | | 15.16 |
|  | C_12 | 7.20 | 0.10 | | 6.60 | | 0.20 | | 7.64 | | 0.11 | | 5.70 | | 10.30 | | 14.35 |
|  | T-I_13 | 5.18 | 0.02 | | 3.65 | | 0.25 | | 5.25 | | 0.09 | | 3.20 | | 7.79 | | 17.98 |
|  | T-II_13 | 4.74 | 0.56 | | 4.85 | | 0.65 | | 5.14 | | 0.08 | | 3.40 | | 7.15 | | 15.22 |
|  | C_13 | 6.85 | 0.15 | | 5.80 | | 0.30 | | 6.72 | | 0.09 | | 4.95 | | 9.27 | | 13.66 |
| NGS_m_ | T-I_11 | 15.50 | 0.10 | | 10.90 | | 1.50 | | 14.95 | | 0.31 | | 7.50 | | 20.90 | | 20.87 |
|  | C_11 | 18.80 | 0.40 | | 12.60 | | 0.60 | | 17.56 | | 0.33 | | 8.70 | | 22.80 | | 18.91 |
|  | T-II_12 | 18.10 | 1.40 | | 14.60 | | 0.10 | | 16.78 | | 0.31 | | 8.65 | | 24.80 | | 18.73 |
|  | C_12 | 20.50 | 1.00 | | 17.70 | | 0.35 | | 21.87 | | 0.33 | | 12.15 | | 28.55 | | 15.17 |
|  | T-I_13 | 14.65 | 0.15 | | 11.80 | | 0.85 | | 15.34 | | 0.28 | | 9.30 | | 21.85 | | 18.06 |
|  | T-II_13 | 11.70 | 0.40 | | 10.90 | | 1.30 | | 13.00 | | 0.22 | | 8.80 | | 18.95 | | 17.01 |
|  | C_13 | 20.35 | 0.85 | | 15.40 | | 0.05 | | 19.84 | | 0.32 | | 12.40 | | 26.13 | | 15.88 |
| GWS_m_ (g) | T-I_11 | 0.65 | 0.04 | | 0.39 | | 0.13 | | 0.61 | | 0.01 | | 0.30 | | 0.90 | | 22.42 |
|  | C_11 | 0.89 | 0.04 | | 0.59 | | 0.03 | | 0.79 | | 0.02 | | 0.44 | | 1.11 | | 22.53 |
|  | T-II_12 | 0.72 | 0.06 | | 0.56 | | 0.04 | | 0.61 | | 0.01 | | 0.37 | | 0.94 | | 20.24 |
|  | C_12 | 0.86 | 0.04 | | 0.71 | | 0.02 | | 0.99 | | 0.01 | | 0.64 | | 1.23 | | 14.18 |
|  | T-I_13 | 0.65 | 0.01 | | 0.43 | | 0.05 | | 0.66 | | 0.02 | | 0.35 | | 1.04 | | 22.77 |
|  | T-II_13 | 0.54 | 0.04 | | 0.45 | | 0.07 | | 0.53 | | 0.01 | | 0.34 | | 0.83 | | 19.78 |
|  | C_13 | 0.97 | 0.04 | | 0.70 | | 0.01 | | 0.93 | | 0.02 | | 0.63 | | 1.39 | | 17.81 |
| LS_l_ (cm) | T-I_11 | 4.90 | 0.10 | | 4.05 | | 0.65 | | 5.52 | | 0.10 | | 3.40 | | 7.88 | | 17.52 |
|  | C_11 | 6.00 | 0.30 | | 4.57 | | 0.27 | | 5.96 | | 0.09 | | 3.70 | | 8.32 | | 15.67 |
|  | T-II_12 | 5.95 | 0.05 | | 3.50 | | 0.70 | | 5.56 | | 0.09 | | 3.65 | | 7.80 | | 15.43 |
|  | C_12 | 6.10 | 0.10 | | 5.70 | | 0.20 | | 6.56 | | 0.09 | | 5.10 | | 8.80 | | 13.26 |
|  | T-I_13 | 4.49 | 0.09 | | 3.00 | | 0.20 | | 4.59 | | 0.08 | | 2.65 | | 6.75 | | 18.18 |
|  | T-II_13 | 4.00 | 0.10 | | 3.15 | | 0.25 | | 4.27 | | 0.07 | | 1.75 | | 5.80 | | 15.98 |
|  | C_13 | 5.20 | 0.20 | | 4.75 | | 0.35 | | 5.61 | | 0.08 | | 3.90 | | 8.12 | | 14.98 |
| NGS_l_ | T-I_11 | 13.22 | 1.08 | | 8.70 | | 1.30 | | 11.43 | | 0.24 | | 5.86 | | 16.47 | | 20.58 |
|  | C_11 | 14.49 | 0.09 | | 10.89 | | 1.09 | | 13.75 | | 0.28 | | 6.45 | | 19.00 | | 20.26 |
|  | T-II_12 | 16.05 | 0.65 | | 8.40 | | 1.00 | | 15.20 | | 0.29 | | 8.35 | | 20.60 | | 19.19 |
|  | C_12 | 17.75 | 0.15 | | 15.55 | | 1.05 | | 19.13 | | 0.30 | | 11.10 | | 25.60 | | 15.85 |
|  | T-I_13 | 12.41 | 0.01 | | 9.55 | | 0.55 | | 12.25 | | 0.23 | | 7.00 | | 17.65 | | 18.80 |
|  | T-II_13 | 10.02 | 0.52 | | 7.30 | | 0.70 | | 8.96 | | 0.20 | | 2.70 | | 13.45 | | 21.83 |
|  | C_13 | 16.70 | 0.10 | | 12.60 | | 0.40 | | 16.20 | | 0.25 | | 10.00 | | 22.64 | | 15.52 |
| GWS_l_ (g) | T-I_11 | 0.51 | 0.04 | | 0.31 | | 0.11 | | 0.41 | | 0.01 | | 0.19 | | 0.63 | | 22.22 |
|  | C_11 | 0.59 | 0.00 | | 0.39 | | 0.08 | | 0.52 | | 0.01 | | 0.30 | | 0.77 | | 21.87 |
|  | T-II_12 | 0.60 | 0.02 | | 0.30 | | 0.07 | | 0.52 | | 0.01 | | 0.29 | | 0.80 | | 20.76 |
|  | C_12 | 0.74 | 0.02 | | 0.61 | | 0.07 | | 0.84 | | 0.01 | | 0.47 | | 1.11 | | 16.72 |
|  | T-I_13 | 0.54 | 0.01 | | 0.37 | | 0.01 | | 0.50 | | 0.01 | | 0.24 | | 0.81 | | 20.67 |
|  | T-II_13 | 0.37 | 0.03 | | 0.23 | | 0.01 | | 0.33 | | 0.01 | | 0.09 | | 0.53 | | 24.06 |
|  | C_13 | 0.75 | 0.01 | | 0.39 | | 0.03 | | 0.71 | | 0.01 | | 0.42 | | 1.08 | | 18.61 |
| TGW  (g) | T-I_11 | 39.69 | 1.11 | | 31.29 | | 3.66 | | 37.48 | | 0.49 | | 25.12 | | 46.46 | | 12.95 |
|  | C_11 | 41.65 | 1.37 | | 42.61 | | 0.73 | | 40.91 | | 0.55 | | 26.62 | | 53.37 | | 13.33 |
|  | T-II_12 | 38.52 | 0.12 | | 36.96 | | 0.14 | | 35.59 | | 0.46 | | 25.90 | | 44.88 | | 12.95 |
|  | C_12 | 41.82 | 0.31 | | 39.57 | | 0.89 | | 44.82 | | 0.35 | | 36.39 | | 53.94 | | 7.72 |
|  | T-I_13 | 43.64 | 0.47 | | 37.11 | | 0.33 | | 41.68 | | 0.33 | | 31.74 | | 48.42 | | 7.95 |
|  | T-II_13 | 41.71 | 0.74 | | 36.98 | | 0.25 | | 39.23 | | 0.29 | | 30.08 | | 46.17 | | 7.44 |
|  | C_13 | 46.36 | 0.04 | | 38.91 | | 0.88 | | 45.31 | | 0.27 | | 38.41 | | 51.82 | | 5.88 |
| GWP  (g) | T-I_11 | 1.64 | 0.07 | 1.21 | | 0.59 | | 1.43 | | 0.03 | | 0.67 | | 2.11 | | 20.86 | |
|  | C_11 | 2.71 | 0.21 | 1.71 | | 0.14 | | 2.01 | | 0.03 | | 1.08 | | 2.71 | | 17.32 | |
|  | T-II_12 | 2.40 | 0.01 | 1.24 | | 0.01 | | 2.36 | | 0.04 | | 1.28 | | 3.27 | | 15.73 | |
|  | C_12 | 3.44 | 0.40 | 2.67 | | 0.20 | | 3.26 | | 0.04 | | 1.95 | | 4.05 | | 12.71 | |
|  | T-I_13 | 2.20 | 0.13 | 1.48 | | 0.02 | | 2.10 | | 0.03 | | 1.43 | | 2.83 | | 15.72 | |
|  | T-II_13 | 1.82 | 0.03 | 1.13 | | 0.07 | | 1.47 | | 0.03 | | 0.88 | | 2.15 | | 19.75 | |
|  | C_13 | 3.11 | 0.14 | 1.96 | | 0.11 | | 3.05 | | 0.04 | | 2.03 | | 4.34 | | 12.98 | |
| HD  (days) | T-I_11 | 51.00 | 0.00 | 43.00 | | 0.00 | | 51.98 | | 0.59 | | 40.00 | | 65.00 | | 11.42 | |
|  | C_11 | 51.50 | 0.50 | 42.00 | | 0.00 | | 50.88 | | 0.57 | | 39.50 | | 63.00 | | 11.13 | |
|  | T-II_12 | 60.00 | 0.00 | 46.00 | | 0.00 | | 56.77 | | 0.31 | | 48.00 | | 62.50 | | 5.42 | |
|  | C_12 | 55.00 | 0.00 | 42.00 | | 0.00 | | 53.95 | | 0.53 | | 41.00 | | 61.00 | | 9.83 | |
|  | T-I_13 | 54.00 | 0.00 | 38.00 | | 0.00 | | 51.69 | | 0.55 | | 39.00 | | 58.00 | | 10.69 | |
|  | T-II_13 | 55.00 | 0.00 | 42.00 | | 0.00 | | 50.12 | | 0.32 | | 43.00 | | 56.00 | | 6.36 | |
|  | C_13 | 52.50 | 0.50 | 36.00 | | 1.00 | | 47.58 | | 0.56 | | 36.00 | | 53.50 | | 11.71 | |
